# Phytochemical Characterization and Anti-*Helicobacter pylori* Potential of *Haloxylon articulatum* Extracts: Antioxidant Activity and Molecular Docking Insights

**DOI:** 10.3390/molecules30173520

**Published:** 2025-08-28

**Authors:** Reyadh Al-Rashidi, Hana Nasrallah, Amal Bouazzi, Amira Zaïri

**Affiliations:** 1 Department of Basic Science, College of Dentistry, University of Kut, Wasit 52001, Iraq; reyadh.radhi@alkutcollege.edu.iq; 2 Department of Biochemistry, Faculty of Medicine, University of Sousse, Sousse 4002, Tunisia; hananasrallah.hn@gmail.com; 3 Department of General Surgery, Sahloul University Hospital, Medicine Faculty of Sousse, University of Sousse, Sousse 4002, Tunisia; amal_bouazzi@yahoo.fr

**Keywords:** *Haloxylon articulatum*, antioxidant activity, *Helicobacter pylori*, antibacterial activity, molecular docking, ADMET analysis, LC-HRMS/MS

## Abstract

*Haloxylon articulatum* is traditionally used for treating infections, digestive issues, and oxidative stress. Despite its ethnopharmacological relevance, its phytochemistry and biological activities, particularly in Iraq, are underexplored. This study investigated the phytochemical composition of *H. articulatum* extracts and evaluated their antioxidant and anti-*Helicobacter pylori* activities, supported by molecular docking and in silico ADMET analysis. Methanol/water and ethyl acetate extracts from roots and aerial parts were analyzed using LC-HRMS/MS. Antioxidant capacity was measured via DPPH assay, and anti-*H. pylori* activity was assessed using broth microdilution. Molecular docking targeted bacterial isoleucyl-tRNA synthetase, and ADMET predictions were carried out with SwissADME and ADMETlab. Phytochemical profiling identified 32 compounds, including phenolamides, flavonoids, alkaloids, and triterpenoid glycosides. Root extracts exhibited stronger antioxidant and antibacterial effects than aerial parts. Ethyl acetate extracts were inactive. Phenolamides, N-caffeoyltyramine, and sinapoyltyramine, present in the extract, showed significant activity (MICs = 54 ± 0.92 and 74 ± 1.05 µg/mL). Docking supported their strong binding to the target enzyme. ADMET results indicated good oral bioavailability and low toxicity. This study is the first to report the anti-*H. pylori* activity of *H. articulatum* and to characterize its Iraqi chemotype through advanced metabolomics. The findings highlight the plant’s potential as a source of multifunctional phytochemicals with antioxidant and antibacterial applications, warranting further preclinical development and toxicological investigation.

## 1. Introduction

*Haloxylon articulatum*, commonly known as the salt tree or white saxaul, is a perennial shrub of the Amaranthaceae family, widely distributed across arid and semi-arid regions of North Africa, the Middle East, and Central Asia. Traditionally, it has been used in local pharmacopoeias to treat a wide range of ailments, including diabetes, infections, and inflammatory disorders. Beyond its medicinal applications, the plant plays an essential ecological role in combating desertification and improving soil stability in degraded environments [1,2]. Taxonomically, *H. articulatum* is also recognized under various synonyms such as Haloxylon scoparium Pomel, Hammada scoparia (Pomel) Iljin, and Arthrophytum scoparia (Pomel) Iljin [3]. Despite its ecological importance and traditional use, scientific research on *Haloxylon articulatum* remains limited, particularly when compared to other desert plants with similar applications. Most published studies have focused on general phytochemical screening and antioxidant activity [4,5,6], leaving a significant gap in understanding the molecular and pharmacological basis of its bioactivity. This underexploration highlights the need for further investigation to validate and potentially valorize its medicinal potential.

Phytochemical investigations have revealed that *H. articulatum* contains several classes of bioactive secondary metabolites, including alkaloids, flavonoids, tannins, saponins, and phenolic acids, particularly in its aerial parts [5,6,7]. Notably, Pallares et al. identified up to 18 phenolic compounds in *H. scoparium*, including hydroxycinnamic acids and flavonoid derivatives [4]. Other related species, such as H. salicornicum and *H. ammodendron*, have shown antimicrobial, antioxidant, and anticancer properties, indicating a promising pharmacological potential across the genus [8,9].

The biological activities of *H. articulatum* extracts, particularly antioxidant, antibacterial, antifungal, and antidiabetic effects, are influenced by the extraction method and solvent system used. Innovative techniques like pulsed electric field extraction and microwave-assisted extraction have demonstrated improved yields of polyphenols and enhanced bioactivity [9,10]. For example, ethyl acetate and butanol fractions of *H. scoparium* flowers exhibited strong antioxidant activity [10], while ethanolic extracts of *H. ammodendron* were active against Gram-positive bacteria such as Staphylococcus aureus and Streptococcus mutans [11]. Among the isolated compounds, N-caffeoyltyramine and sinapoyltyramine have drawn attention for their antiproliferative, antibacterial, and antioxidant properties. N-caffeoyltyramine has been shown to modulate protein tyrosine kinase activity and enhance hepatic lipid metabolism without observable toxicity [12,13,14].

*Helicobacter pylori*, a Gram-negative bacterium linked to chronic gastritis, peptic ulcer disease, and gastric carcinoma, remains a major global health concern. The rising prevalence of antibiotic-resistant *H. pylori* strains, particularly to metronidazole, calls for the development of new therapeutic agents [15,16]. In Iraq, the infection rate among dyspeptic patients exceeds 60% [15], yet no studies have evaluated the potential of *H. articulatum* against *H. pylori*. Moreover, data from Iraq remain scarce overall, despite several investigations conducted in Algeria, Tunisia, Morocco, and Portugal [2,4,5,6,17,18].

Given the scarcity of targeted pharmacological studies, especially concerning *H. pylori*, the present study seeks to fill this gap. We aim to explore the phytochemical profile, antioxidant potential, and antibacterial activity of *H. articulatum* extracts, with particular emphasis on anti-*H. pylori* activity. Furthermore, molecular docking and ADMET (Absorption, Distribution, Metabolism, Excretion, and Toxicity) analyses of N-caffeoyltyramine and sinapoyltyramine were performed to evaluate their pharmacokinetic and safety profiles as candidate antibacterial agents.

## 2. Results

### 2.1. Phytochemical Analysis of the Haloxylon Articulatum Extract

The LC-HRMS/MS analysis of aerial and root extracts of *Haloxylon articulatum* revealed a complex metabolite profile comprising a total of 32 compounds across various chemical classes. Table 1 summarizes the tentative identifications (Id.), chemical classification, retention times (TRs), molecular formulas (MFs), ion types (adduct), observed and calculated *m*/*z* values, mass accuracy (Δ, ppm), and key fragment ions (MS/MS). Compound identification was achieved by matching the accurate mass measurements and fragmentation profiles with reference spectra from databases such as PubChem, METLIN, KNApSacK, and the NIST Chemistry WebBook.

The phytochemical analysis indicated the presence of alkaloids, flavonoid glycosides, phenolic derivatives, saccharides, fatty acid derivatives, and triterpenoid glycosides. Among the detected alkaloids, N-methylisosalsoline ([M+H]^+^, *m*/*z* 208.1357, TR 2.01 min) and carnegine ([M+H]^+^, *m*/*z* 222.1501, TR 2.51 min) were identified, both belonging to the tetrahydroisoquinoline subclass and exhibiting diagnostic fragments such as *m*/*z* 191, 165, and 145. A β-carboline derivative, tetrahydroharmane ([M+H]^+^, *m*/*z* 187.1221, TR 4.26 min), was also identified, characterized by MS/MS fragments at *m*/*z* 170, 158, and 144. Notably, N-methyltryptamine ([M+H]^+^, *m*/*z* 175.1222, TR 2.84 min) was exclusively detected in aerial tissues, while all other alkaloids were present in both aerial and root extracts. A diverse range of flavonoid glycosides was also detected, but only in aerial parts of the plant. These included naringin ([M−H]^−^, *m*/*z* 579.1712, TR 6.25 min), which displayed characteristic fragments at *m*/*z* 419, 289, 271, and 151; quercetin deoxyhexosyl-hexoside ([M−H]^−^, *m*/*z* 609.1428, TR 9.42 min); and isorhamnetin dihexoside ([M−H]^−^, *m*/*z* 639.1552, TR 6.59 min). These compounds were identified based on fragmentation patterns reflecting neutral loss of sugar moieties and the presence of diagnostic aglycone ions. The detection of isorhamnetin deoxyhexosyl-deoxyhexosyl-hexoside ([M−H]^−^, *m*/*z* 769.2185, TR 9.00 min) further illustrated the structural diversity of glycosylated flavonoids.

Phenolic acids and their derivatives were also abundant. Ferulic acid ([M−H]^−^, *m*/*z* 193.0488, TR 10.41 min) was identified by characteristic fragments at *m*/*z* 161 and 133 and was exclusively found in root extracts. In contrast, N-caffeoyltyramine ([M−H]^−^, *m*/*z* 298.1055, TR 11.08 min), feruloyltyramine ([M−H]^−^, *m*/*z* 312.1225, TR 14.67 min), and sinapoyltyramine ([M−H]^−^, *m*/*z* 342.1316, TR 15.17 min) were present in both aerial and root parts. These compounds exhibited fragmentation patterns consistent with typical phenolamide profiles, including ions at *m*/*z* 190, 178, and 135.

The presence of triterpenoid glycosides was prominent, particularly in root extracts. Notable examples include achyranthoside E ([M−H]^−^, *m*/*z* 925.4453, TR 25.08 min) and compounds putatively assigned as triterpenoids or triterpenoid glycosides (e.g., [M−H]^−^, *m*/*z* 925.4801, TR 24.25 min; [M−H]^−^, *m*/*z* 1087.5012, TR 23.67 min).

Fatty acid derivatives and sphingolipids were also detected. Hydroxyoctadecadienoic acid ([M−H]^−^, *m*/*z* 295.225, TR 34.92 min) and trihydroxyoctadecadienoic acid ([M−H]^−^, *m*/*z* 327.2151, TR 18.67 min) were among the most abundant, along with phytosphingosine ([M+H]^+^, *m*/*z* 318.2998, TR 27.93 min), a bioactive sphingolipid commonly found in plant tissues. All features with adequate signal intensity were characterized based on molecular formulas and MS/MS fragmentation patterns, as listed in Table 1. Overall, these findings highlight *Haloxylon articulatumas* as a rich source of diverse secondary metabolites with promising pharmacological potential.

### 2.2. Determination of the Antioxidant Activity Based on the DPPH Free Radical Scavenging Activity Method

The extraction process resulted in two distinct dark brown extracts, between which the first extract was from the aerial parts, whereas the other one was extracted from the roots of *H. articulatum*. The extraction yields were 15% in the case of root parts and 10% for the aerial parts. The methanol/water extract from the root parts exhibited a higher DPPH radical scavenging activity (82.0%) compared to the extract retrieved from the aerial parts of the plant (58.7%). These results indicate that the root extract has a stronger antioxidant potential than the aerial-part extract under the tested conditions. The DPPH assay confirmed that the *Haloxylon articulatum* extracts enable significant free radical scavenging activity, which eventually supports the potential of Haloxylon species as natural sources of antioxidants.

### 2.3. Antibacterial Activities of Haloxylon Extracts on Helicobacter Pylori (H. pylori)

The methanol/water extracts of the *H. articulatum* (both root and aerial parts) were successfully dissolved without precipitation. This phenomenon confirms the extract’s solubility in respective solvents at varying concentrations (i.e., 10–200 µg/mL). However, due to low polarity, the ethyl acetate extract of the specimen *H. articulatum* (root and aerial parts) was found to be less soluble than methanol/water. The final four samples tested are presented below. The results of the MIC are depicted in Table 2.

Table 2 presents the MIC values, indicating that S1 (root extract in methanol/water) exhibited the lowest MIC value (95 µg/mL) and demonstrating the strongest antibacterial activity against *H. pylori*. In comparison, S2 (aerial part extract) also showed notable activity with an MIC of 116 µg/mL, though it was less potent than S1. In contrast, the ethyl acetate extracts displayed weak antibacterial effects, requiring significantly higher concentrations to inhibit bacterial growth. These results highlight the superior efficacy of the methanol/water extraction system, particularly for the root extract, and suggest that the active antibacterial compounds in *H. articulatum* are likely polar in nature. N-caffeoyltyramine and sinapoyltyramine, present in the extract, were used as reference standards to compare biological activity, and their respective MIC values are detailed in Table 3.

### 2.4. Molecular Docking

This study employed AutoDock Vina 1.2.0 to perform a molecular docking investigation on the *H. pylori* isoleucyl-tRNA synthetase (PDB ID: 8WNJ), obtained from the Protein Data Bank (https://www.rcsb.org/structure/4y6m (accessed on 7 March 2025)) [19]. The crystal structure of the *H. pylori* isoleucyl-tRNA synthetase was meticulously prepared using AutoDockTools 1.5.6. This process involved the removal of water molecules, incorporation of polar hydrogen atoms, and assigning the Gasteiger charges to ensure accurate representation of the protein’s electrostatic properties. The ligands were generated and optimized using OpenBabel 3.1.1, generating 3D structures with minimized energy states [19,20].

**Figure 1:**
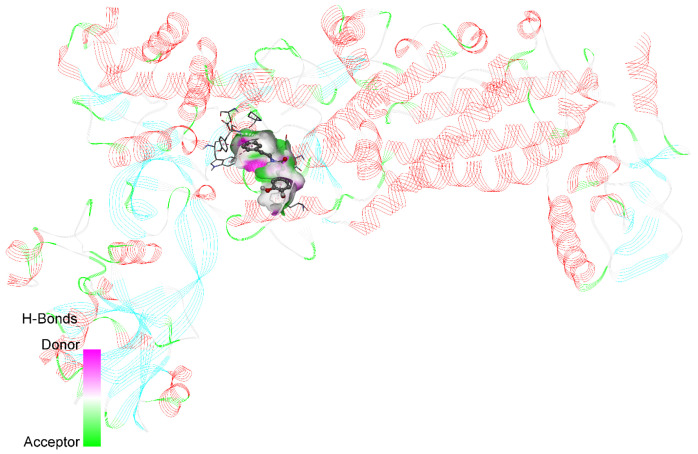
3D ribbon representation of native ligand with *H. pylori* isoleucyl-tRNA synthetase (PDB ID: 8WNJ).

In order to enhance the precision of the docking process, the authors carefully defined the grid box centered on the active site at coordinates such as X: 9.052774, Y: 16.410774, and Z: −1.715387. The PyRx virtual screening tool was utilized, in which all the ligands were fed as input, including the native ligand, and the docking assays were performed. This approach allowed for a comprehensive evaluation of the potential binding modes and affinities. The resulting poses were meticulously analyzed based on the binding affinity scores and protein–ligand interactions, with the highest-scoring conformations selected for further investigation. To visualize and interpret the docking results, Biovia Discovery Studio was utilized, which enabled a detailed examination of the molecular interactions between the ligands and the *H. pylori* isoleucyl-tRNA synthetase. Figure 1 shows a 3D ribbon representation of the enzyme complexed with its native ligand, which offers a clear visual understanding of the binding mode and key interactions. This rigorous molecular docking approach provides valuable insights about the potential binding mechanisms of various ligands to the *H. pylori* isoleucyl-tRNA synthetase. Thus, it lays a solid foundation for the structure-based drug design and the development of novel *H. pylori* isoleucyl-tRNA synthetase inhibitors.

The native ligand interacts with several amino acid residues such as GLU569, ASP572, GLY56, ILE603, MET611, GLN573, PRO58, and HIS600. It formed electrostatic attractive charge interactions with GLU569 and ASP572 and multiple hydrogen bonds (both conventional and carbon hydrogen bonds) with multiple residues. N-caffeoyltyramine exhibited a higher binding affinity compared to the native ligand, while its respective docking scores were −9.4 kcal/mol and −8.4 kcal/mol. It formed hydrogen bonds with ASP96 and GLY570 and a carbon hydrogen bond with GLU569.

Additionally, it was also engaged in hydrophobic interactions, including Pi–Pi stacked interactions with PHE602 and TRP541, a Pi–Pi T-shaped interaction with TYR59, and a Pi–Alkyl interaction with PRO57. Sinapoyltyramine secured a docking score of −8.4 kcal/mol, similar to the native ligand. It formed multiple hydrogen bonds with GLN573 and ILE603 and carbon hydrogen bonds with ASN71, ILE603, and GLY67. It further exhibited diverse interactions, including a Pi–Anion electrostatic interaction with ASP572, a Pi–Sulfur interaction with MET611, a Pi–Pi stacked interaction with HIS65, and Pi–Alkyl interactions with ILE603 and VAL618. Both the compounds exhibited different interaction patterns compared to the native ligand, which suggests their potentials for competitive binding. The higher binding affinity of N-caffeoyltyramine indicates its eligibility to be a promising candidate for inhibiting *H. pylori* isoleucyl-tRNA synthetase. However, the diverse interaction profile of sinapoyltyramine, including its electrostatic and hydrophobic interactions, could also contribute to its effectiveness as a potential inhibitor.

**Figure 2:**
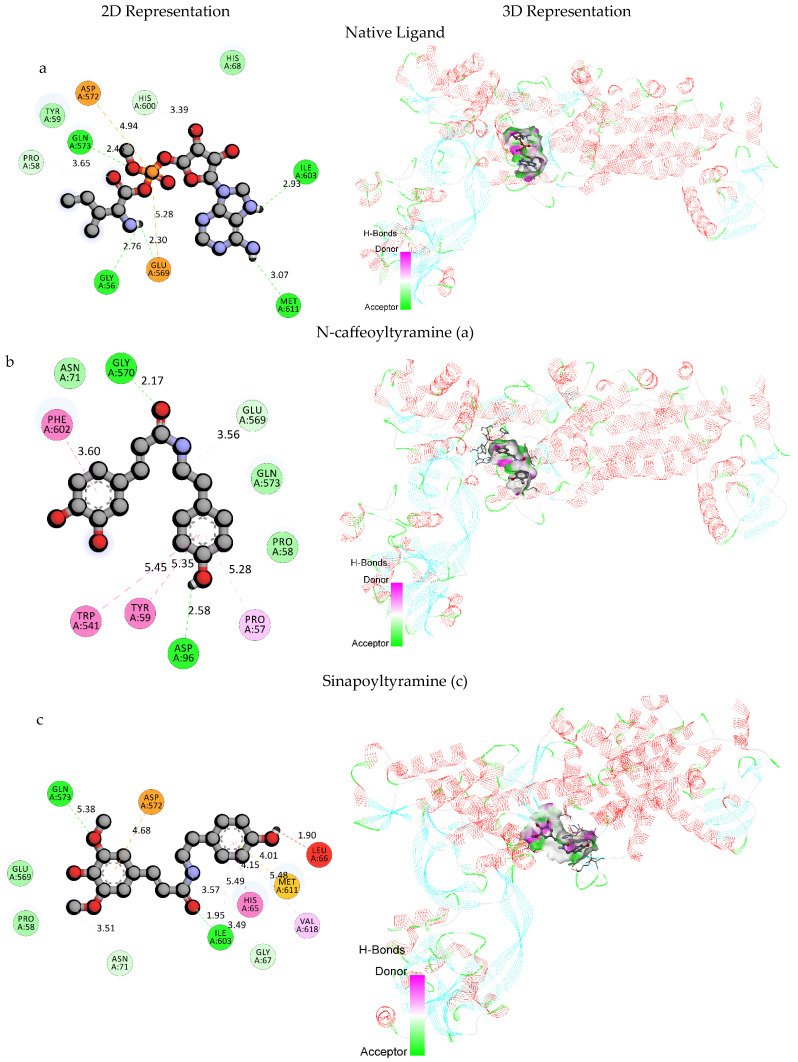
2D and 3D representations of selected compounds (**a**) with native ligand (**b**) N-caffeoyltyramine and (**c**) sinapoyltyramine.

**Figure 3:**
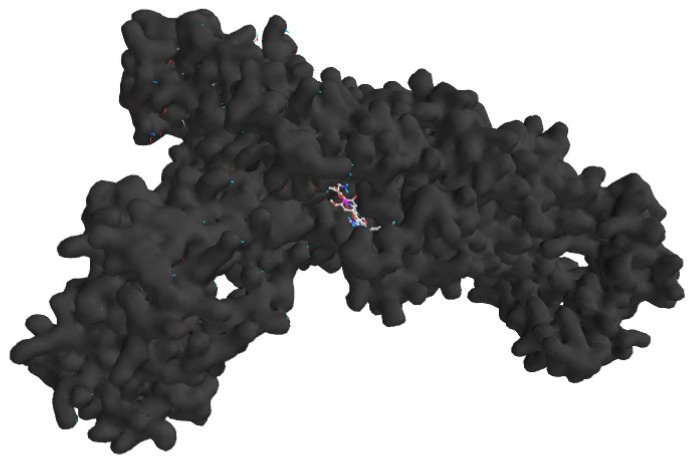
Combined docking poses of the selected compounds and native ligand with *H. pylori* isoleucyl-tRNA synthetase (PDB ID: 8WNJ).

These computational analyses provide insights into the binding mechanisms of N-caffeoyltyramine and sinapoyltyramine, supporting their potential as treatments for *H. pylori* bacterial infections. The binding interactions of the selected derivatives and the native ligand with *H. pylori* isoleucyl-tRNA synthetase (PDB ID: 8WNJ) are depicted in Table 4. The 2D and 3D representations of the selected compounds with native ligands are shown in Figure 2. The combined docking poses of the selected compound and native ligand with *H. pylori* isoleucyl-tRNA synthetase (PDB ID: 8WNJ) are shown in Figure 3.

## 3. Discussion

This study provides the first integrated phytochemical and pharmacological assessment of *Haloxylon articulatum* collected in southern Iraq, using a multifaceted approach involving antioxidant and antibacterial evaluation, high-resolution metabolomic profiling, molecular docking simulations, and in silico ADMET analysis. Our findings highlight the potential of this halophytic species as a promising source of natural compounds with dual antioxidant and anti-*Helicobacter pylori* activity, aligning with its traditional use in folk medicine.

1. Antioxidant Activity: Comparative Efficacy and Phytochemical Correlation

The antioxidant evaluation revealed a potent DPPH radical scavenging activity, with the methanol/water root extract (S1) showing the highest inhibition rate (82%), followed by the aerial part extract (S2). This is consistent with prior reports emphasizing the antioxidant richness of *H. articulatum* roots. For instance, Haida et al. (2020) [20] and Noureddine et al. (2023) [6] observed that methanolic and butanolic extracts of *H. scoparium* roots exhibited stronger DPPH scavenging activity than aerial parts, with IC_50_ values often below 100 µg/mL. The current results extend these findings, confirming that polar extraction (methanol/water) enhances the recovery of phenolic compounds, which are primarily responsible for antioxidant activity [21,22].

The absence of activity in the ethyl acetate fractions (S3 and S4) supports this polarity-dependence hypothesis, as less polar solvents are less effective at extracting flavonoids, phenolic acids, and glycosides. This is in line with previous extractions from other Chenopodiaceae plants where only polar extracts displayed antioxidant properties [23,24].

The LC-HRMS/MS data validated the presence of such compounds, including N-caffeoyltyramine, sinapoyltyramine, ferulic acid, and various flavonoid glycosides, such as quercetin, isorhamnetin, and kaempferol derivatives. These molecules are well-established radical scavengers and synergistic antioxidants, reinforcing the observed biological activity [25,26]. It is worth noting that several of these compounds—particularly the phenolamides—are scarcely reported in Haloxylon species, thus contributing to the novelty of our chemical profile.

2. Antibacterial Potential Against H. pylori: A Novel Finding

The antibacterial assays against *H. pylori* demonstrated significant inhibitory effects for the methanol/water extracts, with MIC values of 95 ± 1.10 µg/mL for the roots and 116 ± 1.95 µg/mL for the aerial parts. This is, to our knowledge, the first report of anti-*H. pylori* activity in *H. articulatum*. While prior studies by Bahri et al. (2020) [7] and Heydarpour Ahvazi et al. (2022) [9] have shown antibacterial effects of *H. scoparium* and *H. ammodendron* against Gram-positive and Gram-negative bacteria, none addressed *H. pylori*, a microaerophilic pathogen of considerable clinical relevance.

The strong activity of the root extract suggests that antimicrobial compounds are more concentrated in the subterranean tissues, likely due to stress-induced secondary metabolism in saline or arid environments. This is supported by traditional uses of *H. articulatum* roots in gastrointestinal ailments and ulcers in Iraqi and North African herbal medicine [27].

Importantly, the MIC values observed here compare favorably with those reported for other natural extracts with anti-*H. pylori* potential, including Nigella sativa, Origanum vulgare, and Glycyrrhiza glabra, whose MICs range between 80 and 250 µg/mL [28,29,30]. Thus, *H. articulatum* extracts appear not only effective but also comparable to well-documented herbal remedies, offering a new therapeutic option, particularly in regions burdened by antibiotic resistance.

3. Bioactive Compounds: Activity and Mechanistic Insight

The tested N-caffeoyltyramine and sinapoyltyramine confirmed their direct contribution to the antibacterial activity, with MICs of 54 ± 0.92 µg/mL and 74 ± 1.05 µg/mL, respectively. These compounds belong to the phenolamide family, which has been increasingly recognized for its antimicrobial and antioxidant roles [31,32]. Prior studies on similar phenolamides from Capsicum annuum and Coffea arabica demonstrated comparable inhibitory effects on bacterial growth and radical scavenging [33,34].

Molecular docking provided additional insight into their mechanism of action. N-caffeoyltyramine showed a stronger binding affinity (−9.4 kcal/mol) to *H. pylori* isoleucyl-tRNA synthetase than the native ligand (−8.4 kcal/mol), forming stable hydrogen bonds and Pi–Pi interactions with critical residues such as GLU569 and PHE602. Sinapoyltyramine also showed favorable interactions, though with a slightly lower affinity (−8.4 kcal/mol). These docking outcomes suggest that both compounds may inhibit bacterial protein biosynthesis by interfering with aminoacylation, which is consistent with the bacteriostatic effect observed.

Interestingly, similar docking behavior and binding affinities have been reported for synthetic oxazolidinones, indicating that these phenolamides might serve as natural scaffolds for developing novel antibacterial drugs [35].

4. Metabolomic Profiling: A Rich and Unusual Phytochemical Spectrum

The untargeted LC-HRMS/MS analysis identified 32 metabolites, revealing a diverse chemical profile not previously reported for Iraqi *H. articulatum*. Along with phenolamides and flavonoids, we identified β-carboline alkaloids (e.g., carnegine and tetrahydroharmane), triterpenoid glycosides (e.g., *achyranthoside E.*), and fatty acid derivatives (e.g., oleamide and ricinoleic acid). The detection of N-methyltryptamine is particularly novel, as this compound is rarely found in desert halophytes and may reflect unique biosynthetic pathways activated under extreme environmental stress.

Compared to earlier GC-MS-based reports, which primarily identified sterols and simple terpenoids, the use of LC-HRMS/MS allowed the identification of less volatile and higher molecular weight compounds [36]. The coexistence of flavonoids, saponins, and alkaloids in the same extract highlights a promising multitarget potential for inflammatory, oxidative, and microbial conditions.

5. In Silico ADMET Prediction and Safety Considerations

ADMET analysis revealed favorable pharmacokinetic properties for both N-caffeoyltyramine and sinapoyltyramine, including high oral bioavailability, good gastrointestinal absorption, and non-hepatotoxicity. These findings are encouraging for future development but should be interpreted cautiously. Although no acute toxicity has been reported for *H. articulatum*, subchronic studies in related species (*H. scoparium*) have shown dose-dependent hepatic and renal effects at concentrations ≥ 500 mg/kg body weight. Hence, experimental toxicological studies, including in vivo genotoxicity and chronic administration, are necessary before clinical translation.

## 4. Materials and Methods

### 4.1. Plant Material Collection and Identification

The entire *Haloxylon articulatum* plant, including aerial parts and roots, was collected in April 2023 from Kut, Wasit Governorate, Iraq (32°29′33.5″ N, 45°51′46.8″ E). The plant was identified by Prof. Dr. Radhi K. Obaid (College of Pharmacy, University of Kut), and a voucher specimen (CPK 189) was deposited in the institutional herbarium. The aerial and root parts were separated, washed thoroughly, air-dried, and ground into fine powder using an electric grinder. The powdered samples were stored at low temperature in the dark until further analysis.

### 4.2. Preparation of Plant Extract Through Hydroalcoholic Maceration

A total of 100 g of each powdered sample was macerated in 500 mL of ethanol/water (70:30, *v*/*v*) to ensure efficient solubilization of both polar and moderately non-polar compounds. The mixture was placed on an orbital shaker at 200 rpm for 24 h at 25 ± 2 °C. After maceration, the extracts were filtered through Whatman No. 1 filter paper, and the filtrates were concentrated under reduced pressure at 40 °C using a rotary evaporator to preserve thermolabile constituents. The extraction yield was calculated as (weight of dried extract/weight of dry plant material) × 100. The resulting extracts were labeled HAAP (aerial parts) and HAR (roots) and stored at 4 °C until further analysis.

### 4.3. Phytochemical Analysis of the Extracts

Total phenolic content (TPC) was determined using the Folin–Ciocalteu method. A 0.5 mL aliquot of each extract was added to a 10 mL flask with 0.5 mL of Folin–Ciocalteu reagent and 1 mL of 20% sodium carbonate. The mixture was diluted to volume with distilled water and incubated in the dark for 60 min. Absorbance was measured at 727 nm using a UV-1650PC Shimadzu spectrophotometer. Gallic acid (5–150 µg/mL) was used to construct a calibration curve. TPC was expressed as mg Gallic Acid Equivalents (GAE/g extract).

### 4.4. Liquid Chromatography High-Resolution Mass Spectrometry/Mass Spectrometry (LC-HRMS/MS)

LC-HRMS/MS analyses were conducted on an Agilent 6540 UHD QTOF mass spectrometer equipped with an ESI source (Agilent Dual Jet Stream). Samples were analyzed in both positive and negative electrospray ionization modes. For each mode, the gas temperature was 350 °C, the nitrogen drying gas flow was 12 L/min, and the nebulizer pressure was 40 psi. The capillary voltage was 4000 V, the skimmer voltage 645 V, and the octopole RF voltage 750 V. Data were acquired over the *m*/*z* 100–1000 range. Continuous infusion of Agilent API TOF reference mix ensured mass accuracy. Spectra were processed with Mass Hunter Qualitative Analysis software (v.B.10.00).

### 4.5. Antioxidant Activity by DPPH Assay

The antioxidant activity was measured using the DPPH radical scavenging method. Extracts (500 μL) at various concentrations (10–100 µg/mL) were mixed with 375 μL ethanol and 125 μL of 0.02% DPPH solution. The mixture was incubated in the dark at room temperature for 60 min. Absorbance was read at 517 nm. Butylated hydroxytoluene (BHT) was used as a positive control. The radical scavenging activity was calculated using the following formula:

% Inhibition = [(A_blank − A_sample)/A_blank] × 100; IC_50_ values were calculated and expressed in µg/mL

### 4.6. Antibacterial Activity Against Helicobacter pylori

#### 4.6.1. Preparation of Extracts

Dried plant powders (10 g) from aerial and root parts were extracted with 100 mL methanol/water (80:20, *v*/*v*) for 24 h at 25–30 °C with shaking. The residue was re-extracted twice. Combined filtrates were concentrated under reduced pressure (40–50 °C) and lyophilized. Ethyl acetate was also used to extract secondary bioactive compounds. Extracts were filtered through 0.22 µm membranes and stored at 4 °C.

#### 4.6.2. Stock Solution Preparation in Methanol: Water

Stock solutions (1 mg/mL) were prepared by dissolving 10 mg of each extract in methanol/water, followed by shaking and sonication for 20 min. Aliquots (10–500 µL) were used to prepare concentrations of 10, 50, 100, 150, 300, and 500 µg/mL.

#### 4.6.3. Stock Solution Preparation in Ethyl Acetate

Same as above, but with ethyl acetate as a solvent. Concentrations ranged from 10 to 200 µg/mL.

#### 4.6.4. Minimum Inhibitory Concentration (MIC) Assay

MICs were determined using the broth microdilution method in 96-well plates, as per Clinical and Laboratory Standards Institute (CLSI) guidelines. Each well contained 90 µL Mueller-Hinton broth and serial dilutions of extracts (50 to 0.049 mg/mL). Then, 10 µL *H. pylori* suspension (0.5 McFarland) was added. Positive controls included bacteria without extract; negative controls used DMSO only. Plates were incubated at 37 °C for 24 h. Then, 25 µL of MTT (0.5 mg/mL) was added to assess bacterial viability. Yellow color indicated inhibition, while purple indicated growth.

### 4.7. Molecular Docking Analysis

Docking was performed using AutoDock Vina 1.2.0 to assess compound binding to EGFR kinase (PDB ID: 1XKK) and *H. pylori* isoleucyl-tRNA synthetase (PDB ID: 8WNJ). Structures were prepared in AutoDockTools 1.5.6 by removing water, adding polar hydrogens, and assigning Gasteiger charges. Ligands were optimized using OpenBabel 3.1.1. For EGFR, the grid box was centered at X: 17.181625, Y: 33.931750, Z: 38.427600. For *H. pylori*, the grid center was X: 9.052774, Y: 16.410774, Z: −1.715387. Docking was run using PyRx, and results were visualized with Biovia Discovery Studio.

### 4.8. In Silico ADMET Analysis

ADMET predictions were performed using SwissADME (http://www.swissadme.ch) and ADMETlab 3.0 (https://admetlab3.scbdd.com/ (accessed on 7 March 2025)). These tools evaluated key pharmacokinetic and toxicity parameters, including oral bioavailability, gastrointestinal (GI) absorption, blood–brain barrier (BBB) penetration, and hepatotoxicity, supporting drug-likeness and lead optimization.

### 4.9. Ethical Considerations

Plant collection complied with institutional and national guidelines. No endangered species were involved.

## 5. Conclusions

This study provides the first integrated pharmacological and metabolomic investigation of *Haloxylon articulatum* from Iraq, revealing its rich phytochemical composition and notable biological potential. The methanol/water extracts, particularly from the roots, exhibited strong antioxidant and antibacterial activities against *Helicobacter pylori*, a pathogen of increasing global concern due to rising antibiotic resistance. The *in vitro* validation of N-caffeoyltyramine and sinapoyltyramine, along with their strong binding affinities in molecular docking simulations, further substantiates their role as key bioactive agents.

The untargeted LC-HRMS/MS profiling identified several classes of compounds—phenolamides, alkaloids, glycosylated flavonoids, and triterpenoid saponins—supporting the plant’s traditional medicinal applications and pointing to multitarget pharmacological mechanisms. The presence of uncommon alkaloids and organ-specific metabolites like N-methyltryptamine adds to the chemotaxonomic and ecological interest of this species.

Importantly, this study introduces *H. articulatum* as a novel candidate for the development of plant-based therapeutic agents targeting *H. pylori*. While in silico ADMET predictions suggest favorable safety profiles, experimental toxicological studies are essential to ensure safe translation into clinical or nutraceutical applications. These findings warrant further investigation, including in vivo pharmacodynamics, bioavailability studies, and formulation development, to fully realize the therapeutic potential of *H. articulatum*.

## Figures and Tables

**Table 1:** Identification of metabolites in the hydroalcoholic extract of *Haloxylon articulatum* by LC-HRMS/MS. “‘+’ indicates presence and ‘−’ absence of the compound in aerial parts or roots.

No	T_R_ (min)	Ion Type	Obs. *m*/*z*	Calc. *m*/*z*	Δ (ppm)	MF	MS/MS	Id.	Class	Aerial Parts	Roots
1	1.26	[M+H]^+^	124.0752	124.0762	8.38	C_7_H_9_NO	118.0293; 115.0375; 109.0503	Anisidine	Anisoles	−	+
2	1.34	[M+Cl]^−^	377.0823	377.0851	7.32	C_12_H_22_O_11_	341.1055; 215.0294; 179.0528	Pentose-Hexose disaccharide	Saccharides	+	+
3	1.59	[M−H]^−^	133.0125	133.0137	9.02	C_4_H_6_O_5_	115.0028; 107.0323	Malic acid	Organic acids	+	+
4	1.84	[M−H]^−^	191.0184	191.0192	4.08	C_6_H_8_O_7_	173.0072; 154.9968; 129.0177; 111.0069	Citric acid	Organic acids	+	+
5	2.01	[M+H]^+^	208.1357	208.1338	−9.37	C_12_H_17_NO_2_	191.1077; 165.0919; 145.0651; 117.0705	N-methylisosalsoline	Tetrahydro-isoquinoline alkaloids	+	+
6	2.51	[M+H]^+^	222.1501	222.1494	−3.15	C_13_H_19_NO_2_	191.1063; 179.1054; 165.0900; 145.0642	Carnegine	Tetrahydro-isoquinoline alkaloids	+	+
7	2.84	[M+H]^+^	175.1222	175.1235	7.54	C_11_H_14_N_2_	144.0787; 132.0797; 127.0533; 117.0657	N-Methyltryptamine	Tryptamine alkaloids	+	−
8	4.26	[M+H]^+^	187.1221	187.1235	7.59	C_12_H_14_N_2_	170.0897; 158.0854; 144.0694; 117.0578	Tetrahydroharmane	β-Carboline Alkaloids	+	+
9	6.25	[M−H]^−^	579.1712	579.1714	0.31	C_27_H_32_O_14_	419.1556; 289.0781; 271.0684; 151.0050	Naringin	Flavonoid glycosides	+	−
10	6.59	[M−H]^−^	639.1552	639.1561	1.44	C_28_H_32_O_17_	476.0937; 313.0295; 245.0878; 168.0010	Isorhamnetin dihexoside	Flavonoid glycosides	+	−
11	7.76	[M−H]^−^	741.1865	741.1878	1.78	C_32_H_38_O_20_	300.0237; 271.0218; 178.9954	Quercetin pentosyl-hexosyl-hexoside	Flavonoid glycosides	+	−
12	9.00	[M−H]^−^	769.2185	769.2191	0.81	C_34_H_42_O_20_	314.0398; 299.0157; 178.9951	Isorhamnetin deoxyhexosyl-deoxyhexosyl-hexoside	Flavonoid glycosides	+	−
13	9.42	[M−H]^−^	609.1428	609.1456	4.51	C_27_H_30_O_16_	343.0396; 300.0227; 271.0198; 150.9986	Quercetin deoxyhexosyl-hexoside	Flavonoid glycosides	+	−
14	10.41	[M−H]^−^	193.0488	193.0501	6.63	C_10_H_10_O_4_	161.0226; 133.0282; 106.0399	Ferulic acid	Phenolic acids and derivatives	−	+
15	10.59	[M−H]^−^	755.197	755.2035	8.55	C_33_H_40_O_20_	314.0264; 271.0076; 178.9810	Quercetin deoxyhexosyl-deoxyhexosyl-hexoside	Flavonoid glycosides	+	−
16	11.08	[M−H]^−^	298.1055	298.1079	8.15	C_17_H_17_NO_4_	256.0964; 178.0478; 135.0418; 107.0475	N-Caffeoyltyramine	Phenolic acids and derivatives	**+**	**+**
17	11.25	[M−H]^−^	609.1424	609.1456	5.17	C_27_H_30_O_16_	357.0560; 315.0452; 271.0189; 150.9983	Quercetin deoxyhexosyl-hexoside	Flavonoid glycosides	+	−
18	11.84	[M−H]^−^	623.1618	623.1612	−0.96	C_28_H_32_O_16_	357.0602; 314.0417; 271.0238; 151.0023	Isorhamnetin deoxyhexosyl-hexoside	Flavonoid glycosides	+	−
19	14.67	[M−H]^−^	312.1225	312.1236	3.46	C_18_H_19_NO_4_	190.0480; 178.0481; 148.0498; 135.0421	Feruloyltyramine	Phenolic acids and derivatives	+	+
20	15.17	[M−H]^−^	342.1316	342.1341	7.42	C_19_H_21_NO_5_	190.0477; 178.0476; 148.0498; 135.0420	Sinapoyltyramine	Phenolic acids and derivatives	**+**	**+**
21	18.67	[M−H]^−^	327.2151	327.2171	6.23	C_18_H_32_O_5_	291.1940; 229.1421; 211.1311; 171.0998	Trihydroxyoctadecadienoic acid	Fatty acids and derivatives	+	+
22	20.42	[M−H]^−^	329.2329	329.2328	−0.33	C_18_H_34_O_5_	293.2117; 229.1434; 211.1333; 171.1016	Trihydroxyoctadecenoic acid	Fatty acids and derivatives	+	+
23	25.08	[M−H]^−^	925.4453	925.4433	−2.16	C_46_H_70_O_19_	793.4320; 485.2209; 381.1891; 130.9821	Achyranthoside E	Triterpenoid glycosides	+	+
24	27.35	[M+H]^+^	274.2719	274.2746	9.84	C_16_H_35_NO_2_	256.2502; 230.2329; 106.0739	Lauryldiethanolamine	Fatty acids and derivatives	−	+
25	27.93	[M+H]^+^	318.2998	318.3008	3.20	C_18_H_39_NO_3_	256.2633; 212.2368; 102.0912	Phytosphingosine	Sphingolipids	−	+
26	29.42	[M+HCOO]^−^	360.2738	360.2750	3.33	C_18_H_37_NO_3_	314.2677; 265.1479; 180.8943	Dehydrophytosphingosine	Sphingolipids	+	+
27	32.17	[M−H]^−^	293.2091	293.2117	8.73	C_18_H_30_O_3_	275.1982; 235.1674; 171.0999; 121.0997	Hydroxyoctadecatrienoic acid	Fatty acids and derivatives	+	+
28	32.18	[M+H]^+^	277.2152	277.2168	5.63	C_18_H_28_O_2_	235.1674; 195.1371; 163.1456; 135.1154	Octadecatetraenoic acid	Fatty acids and derivatives	−	+
29	33.09	[M+H]^+^	280.2632	280.2640	3.00	C_18_H_33_NO	263.2369; 245.2260; 179.1776; 109.1010	Linoleamide	Fatty acids and derivatives	+	+
30	34.75	[M−H]^−^	311.2208	311.2222	4.59	C_18_H_32_O_4_	293.2195; 249.2200	Hydroperoxyoctadecadienoic acid	Fatty acids and derivatives	−	+
31	34.76	[M+H]^+^	279.2319	279.2324	1.83	C_18_H_30_O_2_	261.2204; 209.1529; 137.1319; 123.1161	Linolenic acid	Fatty acids and derivatives	+	+
32	34.92	[M−H]^−^	295.225	295.2273	7.86	C_18_H_32_O_3_	277.2141; 195.1356; 155.1063; 113.0948	Hydroxyoctadecadienoic acid	Fatty acids and derivatives	+	+

**Table 2:** MIC values of *H. articulatum* extracts against *H. pylori*.

Solvent Type	MIC (µg/mL)
S1: Methanol/Water (root)	95 ± 1.10
S2: Methanol/Water (aerial)	116 ± 1.95
S3: Ethyl Acetate (root)	N/A
S4: Ethyl Acetate (aerial)	N/A

**Table 3:** MIC values of the *H. articulatum* samples.

Extract	MIC (µg/mL)
N-Caffeoyltyramine	54 ± 0.92
Sinapoyltyramine	74 ± 1.05

**Table 4:** Binding interactions of the selected derivatives with *H. pylori* isoleucyl-tRNA synthetase.

Amino Acid Residues	Bond Length	Bond Type	Bond Category	Ligand Energy	Docking Score
				(Kcal/mol)	
Native Ligand					
GLU569	5.28248	Electrostatic	Attractive charge	1035.1	−8.4
ASP572	4.94099				
GLY56	2.75667	Hydrogen bond	Conventional Hydrogen bond		
GLU569	2.30041				
ILE603	2.93206				
MET611	3.06697				
GLN573	2.46016				
PRO58	3.64946		Carbon hydrogen bond		
HIS600	3.38735				
N-caffeoyltyramine					
ASP96	2.58434	Hydrogen bond	Conventional Hydrogen bond	129.96	−9.4
GLY570	2.16984				
GLU569	3.55524		Carbon hydrogen bond		
PHE602	3.59624	Hydrophobic	Pi–Pi stacked		
TRP541	5.45397				
TYR59	5.34576		Pi–Pi T-shaped		
PRO57	5.27973		Pi–Alkyl		
Sinapoyltyramine					
GLN573	2.64328	Hydrogen bond	Conventional hydrogen bond	188.58	−8.4
GLN573	2.56585				
ILE603	1.95267				
ASN71	3.50592		Carbon hydrogen bond		
ILE603	3.56813				
GLY67	3.49095				
ASP572	4.67666	Electrostatic	Pi–Anion		
MET611	4.0056	other	Pi–Sulfur		
HIS65	4.14692	Hydrophobic	Pi–Pi stacked		
ILE603	5.49085		Pi–Alkyl		
VAL618	5.47563				

## Data Availability

The authors confirm that the data supporting the findings of this study are available within the article. Raw data that support the findings of this study are available from the corresponding author upon reasonable request.

## Abbreviations

The following abbreviations are used in this manuscript:

ADMET	Absorption, Distribution, Metabolism, Excretion, and Toxicity
TPC	total phenolic content
DPPH	2,2-Diphenyl-1-picrylhydrazyl
BHT	butylated hydroxytoluene
DMSO	dimethyl sulfoxide
MTT	3-(4,5-dimethylthiazol-2-yl)-2,5-diphenyltetrazolium bromide
LC-HRMS/MS	Liquid Chromatography High-Resolution Mass Spectrometry/Mass Spectrometry
MIC	minimum inhibitory concentration
Id	tentative identifications
MF	molecular formulas
TR	retention times
